# McKittrick-Wheelock Syndrome: A Rare Cause of Chronic Diarrhea

**DOI:** 10.7759/cureus.13308

**Published:** 2021-02-12

**Authors:** Abhilasha Jyala, Shehriyar Mehershahi, Niel Shah, Danial H Shaikh, Harish Patel

**Affiliations:** 1 Internal Medicine, BronxCare Health System, Bronx, USA; 2 Gastroenterology, BronxCare Health System, Bronx, USA; 3 Internal Medicine, Bronx-Lebanon Hospital Center, Bronx, USA

**Keywords:** tubulovillous adenoma, mckittrick-wheelock syndrome, chronic diarrhea

## Abstract

Diarrhea is the principal cause of the majority of healthcare utilization. When diarrhea lasts longer than four weeks, it is considered chronic diarrhea. There are several causes of chronic diarrhea, but here we focus on one of the rare causes, known as McKittrick-Wheelock syndrome (MWS). We here present the case of a patient in his seventies with chronic diarrhea, found to have tubulovillous adenoma and diagnosed with McKittrick-Wheelock syndrome. We also discuss the clinical presentation, pathophysiology, and management of MWS.

## Introduction

Diarrhea is a common presentation of gastroenterological diseases. Chronic diarrhea can be defined as increased stool frequency and persistent change of stool consistency (loose stools ranging between types 5 and 7 on the Bristol stool chart) lasting more than four weeks [1]. Common causes include irritable bowel syndrome, inflammatory bowel disease, chronic infections (particularly in immunocompromised patients), or malabsorption syndromes (such as lactose intolerance and celiac disease). Chronic diarrhea can be classified as watery diarrhea, fatty diarrhea, and inflammatory diarrhea. Watery diarrhea can be further classified as secretory and osmotic diarrhea [2]. A rare cause of chronic secretory diarrhea is the McKittrick-Wheelock syndrome (MWS), which is a disorder characterized by hypersecretion of fluid and electrolyte from a rectal tumor; most frequently reported are the villous adenomas. We present a case of an elderly patient with chronic diarrhea who was later diagnosed with MWS.

## Case presentation

A 72-year-old male presented to our emergency department with complaints of worsening chronic diarrhea. The patient had a past medical history of delusional disorder and intermittent chronic diarrhea for the past two years. Surgical history was significant for resection of rectal mass two weeks earlier, which was performed at another institute before the presentation, pathology of which was consistent with villous adenoma. He had no known drug allergy and denied cigarette smoking, recreational drug use, or alcohol use. He also denied any recent travel. The review of the system was otherwise negative. On examination, the patient’s vital signs were within normal limits. Physical signs of dehydration were present; otherwise, the physical exam was unremarkable. 

**Figure 1:**
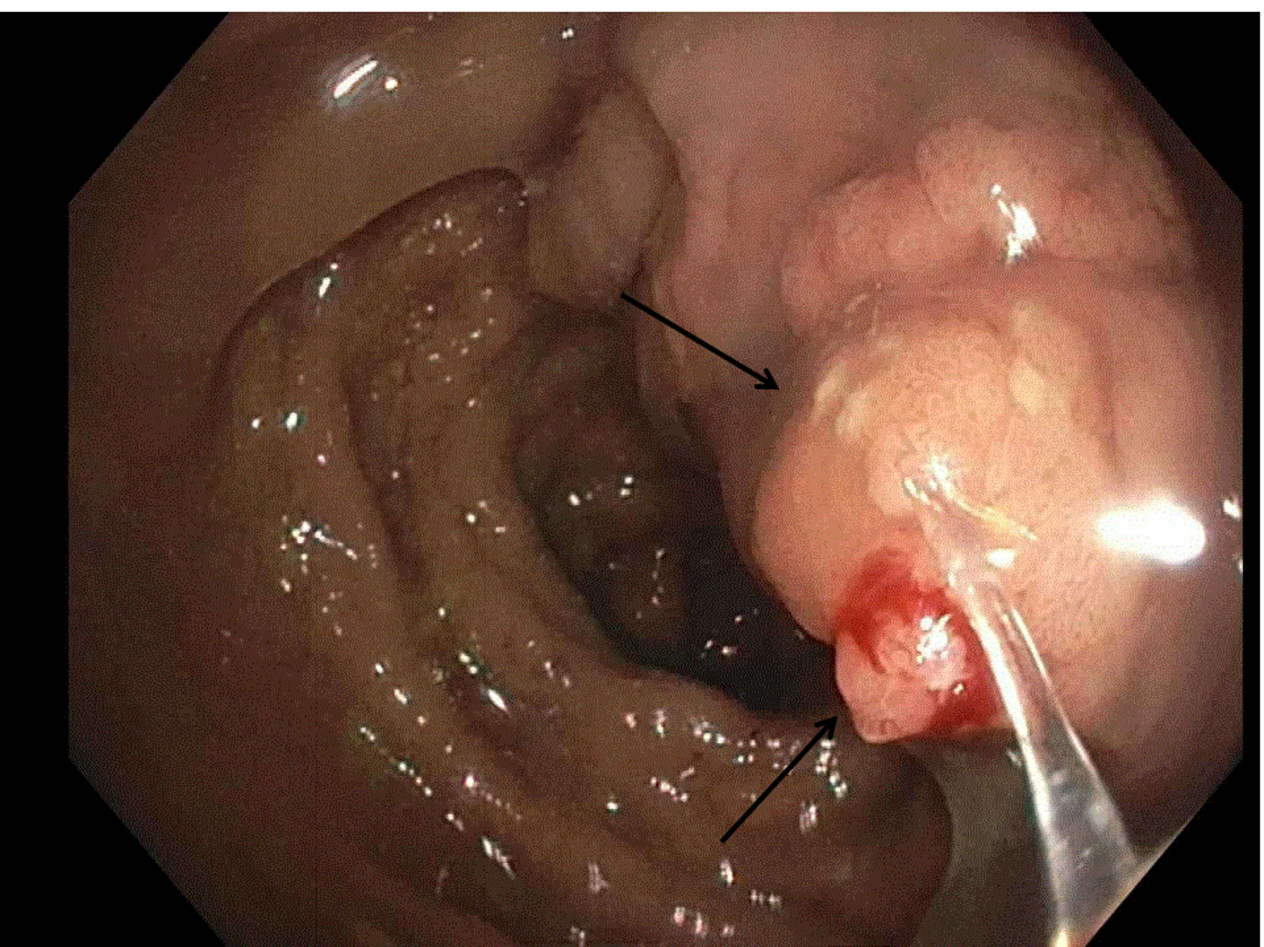
Colonoscopy: recto-sigmoid mass

Laboratory tests were significant for hyponatremia (sodium of 116 mEq/L), hypokalemia (potassium of 2.9 mEq/L), low serum chloride level (52 mEq/L), azotemia (blood urea nitrogen level of 167 mg/dL), elevated creatinine (5 mg/dL), increased anion gap (38 mmoles/L) and elevated blood osmolality (321 mOsm/kg). The stool workup was negative for any infectious cause. Stool osmotic gap consistent with secretory <50 mOsm/kg. Celiac panel and stool occult blood were negative. Erythrocyte sedimentation rate was normal (12 mm/hr). Computerized tomography (CT) scan of the abdomen showed diffuse eccentric rectal wall thickening prominent on the right lateral rectal wall, which was concerning for neoplasm for which patient underwent colonoscopy, which revealed an infiltrative non-obstructing large mass in the recto-sigmoid colon. The mass was circumferential and measured 14 cm in length, extending 2 cms from the anal verge to 16 cms from the anal verge (Figure 1).

**Figure 2:**
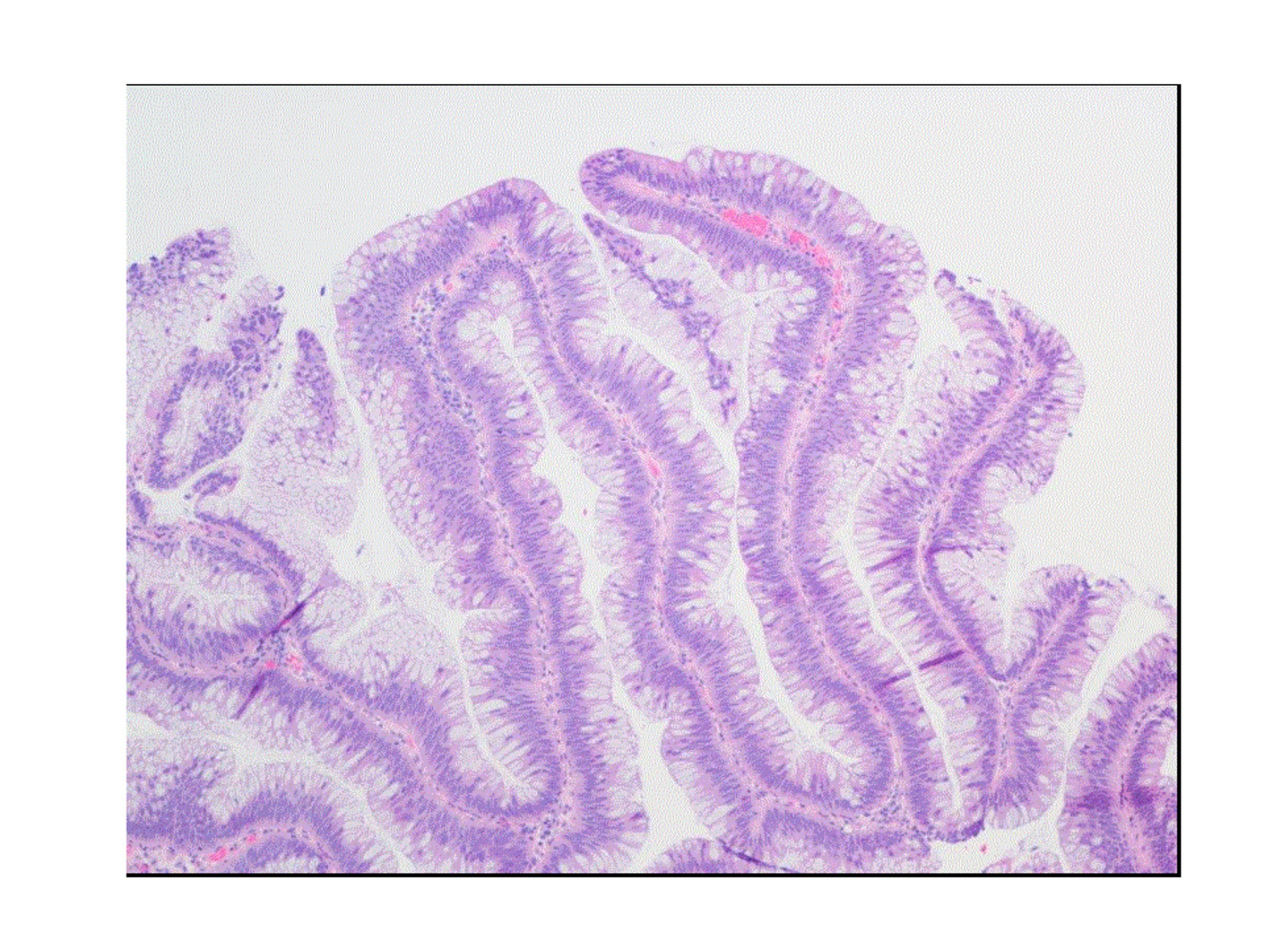
Histology: tubulovillous adenoma

The pathology result was consistent with tubulovillous adenoma (Figure 2). The patient was referred for colorectal surgery and successfully underwent laparoscopic abdominoperineal resection (APR) and loop ileostomy. The surgical specimen pathology report was consistent with tubulovillous adenoma with patchy high-grade dysplasia with no invasion signs. Given the pathology showing villous adenoma, electrolyte abnormalities likely due to secretory diarrhea, and the presence of acute kidney injury, the diagnosis of McKittrick-Wheelock syndrome was made.

## Discussion

McKittrick-Wheelock syndrome (MWS) is a rare disorder of fluid and electrolyte depletion caused by a secretory colorectal tumor, most commonly villous adenoma. This disease was first described by McKittrick and Wheelock in 1954 [3]. MWS is usually characterized by secretory mucinous diarrhea, hyponatremia, hypokalemia, and acute renal failure due to volume depletion. Most patients present with chronic diarrhea and symptoms due to electrolyte imbalances, such as lethargy, muscle cramps, seizures, paresthesia, cramps, ileus, vomiting, hypotension, cardiac arrhythmias, and electrocardiographic changes [4-8]. The incidence and prevalence of MWS are difficult to estimate as some of the cases of MWS have also been reported as electrolyte depletion syndrome [9].

The studies have shown that in patients with villous adenoma, rectal secretions have higher concentrations of prostaglandin E2 (PGE2) and intracellular cyclic adenosine monophosphate (cAMP). PGE2 serves as a secretagogue and thus induces electrolyte efflux (sodium, potassium, and chloride), which is followed by water drawn out of the rectum. Additionally, a large surface area of the villous adenomas further causes increased fluid secretion, which exceeds the reabsorption ability of the remaining normal rectal mucosa, resulting in chronic watery diarrhea. Due to the role of PGE2 in the pathophysiology of MWS, PGE2 synthetase inhibitors (i.e., indomethacin) can be used to decrease stool output in such patients while waiting for surgery [8, 10, 11]. 

The mainstay of treatment is a surgical intervention to remove villous adenoma after correction of fluid and electrolyte imbalances [8]. Brachytherapy and endoscopic tumor resection are alternatives; however, recurrence rates are higher in such interventions [8, 11]. Most colon cancers develop from benign adenomas; particularly, the risk is higher when adenomas are villous and large. Secretory villous adenomas have 100% mortality if left without any treatment [12].

In our case, the patient presented with chronic diarrhea secondary to predominantly villous adenoma in the rectum. As a result of chronic secretory diarrhea, the patient developed electrolyte abnormalities such as hyponatremia, hypochloremia and also developed acute kidney injury likely pre-renal in nature. In our case, the patient already underwent the surgical resection of the rectal mass (completely excised as per pathology report) at another facility before coming to our facility; however, his symptoms were not getting better. CT scan of abdomen and pelvis revealed the presence of rectal mass, which was biopsied during colonoscopy. The pathology showed tubulovillous adenoma. After correcting renal function and electrolyte abnormalities, the patient was referred to colorectal surgery to ensure the complete resection of the mass by low APR and loop ileostomy. 

We think that in our case, the cause of refractory symptoms could be either related to recurrence of the advanced adenoma or missed adenoma on prior colonoscopy due to poor colonic preparation. Additionally, we noticed a change in the histology of resected adenoma from villous adenoma (at another facility) to tubulovillous adenoma (at our facility), which further supports the above-mentioned possibilities. Huang et al. demonstrated that the recurrence rate of advanced adenoma (>1 cm size or tubulovillous/villous histology) was 3.8% during 1-3 years after initial colonoscopy [13]. In general, the recurrence rate of adenoma may be falsely elevated due to missing adenomas during baseline colonoscopy. The colonoscopic miss rate for polyps is about 15-24% [14-16]. The study was conducted to evaluate the recurrence rate of colorectal adenoma prospectively, which showed that the recurrence rate of colorectal adenoma is lower when the miss rate is reduced [17]. Furthermore, the characteristics of adenoma which increase the risk of recurrence include multiple adenomas, large adenomas (>1 cm), or adenomas in the proximal colon [18]. MWS is commonly seen in association with villous adenomas, but there have been few reported cases of tubulovillous adenomas with similar presentation as also seen in our case. However, there is no data available on the prevalence of tubulovillous adenoma in association with MWS currently [8, 9, 19, 20].

## Conclusions

McKittrick-Wheelock syndrome is a rare but life-threatening disorder if not treated. It is usually seen in patients with rectal tumors, most commonly villous adenoma, and is characterized by recurrent diarrhea accompanied by electrolyte disturbances and acute renal failure. The large surface area of the villous adenoma and increased levels of PGE2, which serve as a secretagogue, are responsible for secretory diarrhea and electrolyte disturbances in MWS. The mainstay of the treatment is the correction of electrolyte abnormalities and renal function followed by surgical removal of the adenoma. The trial of PGE2 synthase inhibitor, such as indomethacin, can be given to the patient while waiting for surgery to improve the symptoms. As untreated secretory villous adenoma has 100% mortality, it is very important to diagnose this syndrome in its early phase, and the patient should undergo surgical removal of villous adenoma to improve the prognosis. Missed adenoma during baseline colonoscopy can give a falsely elevated recurrence rate of adenoma that can be reduced by lowering the miss rate by ensuring a careful colonoscopic examination and surveillance.
